# LIVING WITH A ROBOT AT HOME: THE COMPLEXITY OF LIVING WITH ASSISTIVE ROBOTS LABRADOR AND DOUBLE IN EVERYDAY LIFE

**DOI:** 10.1093/geroni/igad104.1353

**Published:** 2023-12-21

**Authors:** Lillian Hung, Donna Case, Haopu Ren, Nathan Velazquez, Olga Petrovskaya

**Affiliations:** University of British Columbia, Vancouver, British Columbia, Canada; University of Michigan Flint, Flint, Michigan, United States; University of British Columbia, Vancouver, British Columbia, Canada; University of Michigan-Flint, Flint, Michigan, United States; University of Victoria, Victoria, British Columbia, Canada

## Abstract

The potential for assistive robots to support older adults’ independence and social connections requires careful consideration of their implications in everyday use. This study investigates the use of two assistive robots, Labrador and Double, in older adults, guided by Actor-Network Theory (ANT). Labrador (a delivery robot) assists with medication management, meals, laundry, house cleaning to support independence, while Double (a teleprence robot) enables virtual social visits. ANT offers a way to understand how the robots interact with different actors, such as older adults, family members, staff, and the environment in which they operate. We applied a qualitative approach to explore how users construct meanings, use, and make sense of the robots in their everyday contexts. Semi-structured interviews and ethnographic fieldwork were conducted with participants to generate data. Reflexive thematic analysis was performed, and three themes emerged: (1) the human-robot relationship, (2) the robot’s agency, and (3) ethical implications. The findings suggest that having the robots in everyday life is a process of constant negotiation with the people, practice, and the robot. The study highlights the challenges and opportunities associated with the implementation of robots to improve quality of life in senior care. While there is a fear that assistive robots will dehumanize caring practices, our study shows that they have the potential to foster innovative user-technology relationships, which requires further research.

